# Molecular testing on bronchial washings for the diagnosis and predictive assessment of lung cancer

**DOI:** 10.1002/1878-0261.12713

**Published:** 2020-06-24

**Authors:** Roberta Roncarati, Laura Lupini, Elena Miotto, Elena Saccenti, Susanna Mascetti, Luca Morandi, Cristian Bassi, Debora Rasio, Elisa Callegari, Valentina Conti, Rosa Rinaldi, Giovanni Lanza, Roberta Gafà, Alberto Papi, Antonio Frassoldati, Silvia Sabbioni, Franco Ravenna, Gian L. Casoni, Massimo Negrini

**Affiliations:** ^1^ Department of Morphology, Surgery and Experimental Medicine University of Ferrara Italy; ^2^ CNR Institute of Genetics and Biomedical Research National Research Council of Italy Milano Italy; ^3^ Azienda Ospedaliero‐Universitaria di Ferrara Division of Respiratory Endoscopy S. Anna Hospital Cona Italy; ^4^ Laboratorio per le Tecnologie delle Terapie Avanzate Tecnopolo University of Ferrara Italy; ^5^ Department of Clinical and Molecular Medicine Sant' Andrea Hospital University "La Sapienza" Rome Italy; ^6^ Pneumology Division State Hospital San Marino Republic of San Marino; ^7^ Division of Anatomic Pathology Carlo Poma Hospital Mantova Italy; ^8^ Azienda Ospedaliero‐Universitaria di Ferrara Division of Anatomic Pathology S. Anna Hospital Cona Italy; ^9^ Department of Medical Sciences University of Ferrara Italy; ^10^ Azienda Ospedaliero‐Universitaria di Ferrara Medical Oncology Unit S. Anna Hospital Cona Italy; ^11^ Department of Life Sciences and Biotechnology University of Ferrara Italy; ^12^ Division of Pneumology and Intensive Respiratory Unit Carlo Poma Hospital Mantova Italy

**Keywords:** early diagnosis, liquid biopsy, lung cancer, molecular test, therapeutic decision‐making

## Abstract

Cytopathological analyses of bronchial washings (BWs) collected during fibre‐optic bronchoscopy are often inconclusive for lung cancer diagnosis. To address this issue, we assessed the suitability of conducting molecular analyses on BWs, with the aim to improve the diagnosis and outcome prediction of lung cancer. The methylation status of *RASSF1A*, *CDH1*, *DLC1* and *PRPH* was analysed in BW samples from 91 lung cancer patients and 31 controls, using a novel two‐colour droplet digital methylation‐specific PCR (ddMSP) technique. Mutations in *ALK*, *BRAF*, *EGFR*, *ERBB2*, *KRAS*, *MAP2K1*, *MET*, *NRAS*, *PIK3CA*, *ROS1* and *TP53* and gene fusions of *ALK*, *RET* and *ROS1* were also investigated, using next‐generation sequencing on 73 lung cancer patients and 14 tumour‐free individuals. Our four‐gene methylation panel had significant diagnostic power, with 97% sensitivity and 74% specificity (relative risk, 7.3; odds ratio, 6.1; 95% confidence interval, 12.7–127). In contrast, gene mutation analysis had a remarkable value for predictive, but not for diagnostic, purposes. Actionable mutations in *EGFR*, *HER2* and *ROS1* as well as in other cancer genes (*KRAS*, *PIK3CA* and *TP53*) were detected. Concordance with gene mutations uncovered in tumour biopsies was higher than 90%. In addition, bronchial‐washing analyses permitted complete patient coverage and the detection of additional actionable mutations. In conclusion, BWs are a useful material on which to perform molecular tests based on gene panels: aberrant gene methylation and mutation analyses could be performed as approaches accompanying current diagnostic and predictive assays during the initial workup phase. This study establishes the grounds for further prospective investigation.

## Graphical abstract

Diagnostic and predictive assessment remains major challenges in lung cancer management. In this work, we investigate the importance of molecular investigations applied to bronchial washings collected during fibre‐optic bronchoscopy for lung lesions. Using a cancer‐specific DNA methylation panel and gene mutations analyses, we demonstrated that bronchial washing represents a useful material for performing molecular tests in lung cancer patients.

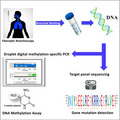

**Abbreviations**
Adks: adenocarcinomas
BAL: bronchoalveolar lavage
BW: bronchial washing
ddMSP: digital methylation‐specific PCR
ddPCR: droplet digital PCR
FOB: fibre‐optic bronchoscopy
LOD: limit of detection
SqK: squamous carcinoma
*T*_a_: annealing temperature

## 1 Introduction

Lung carcinoma is the deadliest cancer worldwide (Siegel *et al*., 2016), with a 5‐year survival rate of 10–15%. Detection of early‐stage tumours is important for reducing mortality. However, most lung cancers are asymptomatic in the early stages, and even in more advanced disease, symptoms are nonspecific and cannot be distinguished from other, nontumour lung diseases (Rivera and Mehta, 2007). Lung cancer is therefore often detected late in its development.

Fibre‐optic bronchoscopy (FOB) is usually the first invasive procedure employed for the diagnosis of lung lesions, allowing physicians to exclude or confirm the presence of synchronous lesions in the airways and other mediastinal structures. Endoscopically, lung cancer lesions can be classified into two categories: visible (central or near‐the‐hilum cancer) and not visible (peripheral bronchoalveolar tumours or paratracheal tumours). FOB allows diagnosis of malignancy in 90% of visible lesions, in 60% of distal lesions and in < 30% of lesions < 2 cm in diameter (Ofiara *et al*., 2012; Thiberville and Salaun, 2010).

Bronchial washing (BW) is a safe, well‐tolerated procedure performed during FOB that allows the harvesting of cytological and histological samples for diagnostic typing. Unfortunately, it is not always possible to reach a definitive diagnosis with this technique, forcing patients to undergo further, potentially more invasive, biopsies.

Several studies have identified lung cancer‐specific DNA alterations in blood, serum, plasma, exhaled breath condensate, bronchoalveolar lavage (BAL) specimens and sputum (Belinsky *et al*., 2005; Han *et al*., 2009; Topaloglu *et al*., 2004). Indeed, aberrant DNA methylation was reported to be a suitable lung cancer biomarker in sputum (Belinsky *et al*., 2006) and in BAL specimens (Topaloglu *et al*., 2004). However, aberrant DNA methylation generally does not affect actionable genes, so the search for clinically actionable alterations has become part of the routine diagnostic workup essential for treatment decisions in lung cancer patients. On this point, liquid biopsy is clinically a highly valuable assay for the assessment of tumour heterogeneity, minimal residual disease and response to therapy in lung cancer (Molina‐Vila *et al*., 2016; Yoneda *et al*., 2019). Analysis of cell‐free DNA for the detection of *EGFR*, *KRAS* or *TP53* mutations in BAL fluid has been reported (Li *et al*., 2014; Park *et al*., 2017). However, current guidelines do not recommend BAL as a routine approach for the diagnosis of peripheral lung lesions, given that the procedure may not be tolerated by all patients and may lead to complications during the process, so impairing the diagnostic efficacy of procedures with higher diagnostic power, such as transbronchial needle aspiration or biopsy. Conversely, BW is a routinely employed, less‐invasive procedure. To our knowledge, there are no studies on the detection of cancer‐gene mutations from BWs.

Thus, we investigated the suitability of using BW fluid in molecular analyses of lung cancer *via* the detection of cancer‐specific alterations of DNA methylation and gene mutations. To this end, we developed a novel, sensitive method based on droplet digital PCR (ddPCR) to detect traces of altered cancer‐specific DNA methylation; given the importance of identifying sensitive and specific diagnostic tools for nonvisible lung lesions, we assessed its ability to detect distal, or peripheral, lesions. Moreover, a panel of 12 cancer genes was used for the identification of actionable mutations, which is needed for therapy selection in advanced lung adenocarcinomas (Adks).

## 2 Methods

### 2.1 Patient cohort

One hundred twenty‐nine consecutive BWs were collected at the University Hospital of Ferrara, Italy, from patients undergoing FOB for suspected lung cancer. Almost all patients were current or former smokers. The study protocol was approved by the local ethics committee and the study methodologies conformed to the standards set by Declaration of Helsinki. All study participants provided a written informed consent for the use of their sample for research purposes. Clinical features of the cohort are reported in Table 1. The 31 controls were defined as benign cases based on several criteria, which included not only negative FOB, but also definitive pathological diagnosis from other more invasive samplings, as well as proven lesion reduction/resolution or lesion stability for at least 12 months upon clinical/radiological follow‐up. Seven metastatic patients were excluded from further statistical analyses and not included in Table 1.

**Table 1:** Clinicopathological features of patients enrolled in the BW study. ND, not determined; NSCLC, nonsmall cell lung cancer; P/Y, packs of cigarettes per year; SCLC, small cell lung cancer.

	Cancer patients	Control patients
Patients		
Total^a^	91	31
Male	60	21
Female	31	10
Median age (range)	71 (47–85)	66 (42–86)
Smoke habits		
Current smoker		
> 10 P/Y	25	8
< 10 P/Y	0	4
Former smoker		
> 10 P/Y	42	11
< 10 P/Y	5	0
Nonsmoker	10	1
Not known	9	7
Diagnosis		
NSCLC: adenocarcinoma	41	
NSCLC: squamous cell carcinoma	32	
SCLC	11	
Lung cancer: undefined	7	
Inflammation		18
Hyperplasia		4
Squamous metaplasia		3
Sarcoidosis		2
Pneumonia		1
Pleurisy		1
Tuberculosis		2
Stage		
1	13	
2	7	
3	25	
4	43	
ND	3	

^a^ Seven patients with metastases were excluded from the analyses.

### 2.2 Fibre‐optic bronchoscopy

During FOB, one or more BW samples were collected for cytological investigation. FOB was performed for diagnostic purposes based on the results of chest computer tomography and/or total body positron emission tomography. The endoscopic procedure was performed by introducing a bronchoscope (FB15V, Fb18V; Pentax Corporation, Tokyo, Japan) nasally while patients were in the supine position. Patients were sedated with midazolam (0.035 mg·kg^−1^ IV, with incremental doses of 1 mg being given as needed), and multiparameter monitoring (pulse oximetry, heart rate, blood pressure) conducted (Contoli *et al*., 2013). Local anaesthesia of the upper and lower respiratory tracts was achieved with lidocaine (10% spray and 2% solution). During all FOBs, BW fluid was collected in a trap by aspiration through the operating channel after instilling 20–40 mL of isotonic saline solution. In the case of visible lesions, the bronchoscope was positioned next to the tumour, wedging the tip of the bronchoscope into the segment where the lesion was located (van der Drift *et al*., 2005; Du Rand *et al*., 2013); here, an endobronchial biopsy was also taken. For nonvisible lesions, transbronchial needle aspiration, transbronchial lung biopsy or both, were performed under fluoroscopic guidance with the C‐arm system (Archovis Ing. Burgatti S.p.A, Bologna, Italy); biopsies obtained from these three procedures and the collected BW samples were eligible for pathological diagnosis.

### 2.3 DNA and RNA isolation

After collection, about 10 mL of the BW samples was immediately centrifuged. Cell pellets were stored at −80°C in the homogenization solution of the Maxwell miRNA Tissue kit (Promega, Madison, WI, USA) for days to a maximum of 8 weeks. DNA and RNA were isolated using the automated Maxwell system (miRNA Tissue kit; Promega), according to the manufacturer's instructions. DNAse was not added during the extraction, allowing us to obtain DNA and RNA from the same starting sample. Nucleic acids were quantified using the Qubit fluorometer (Thermo Fisher Scientific, Waltham, MA, USA).

### 2.4 DNA bisulphite conversion

DNA samples (500 ng) were preliminarily modified by chemical treatment with sodium bisulphite (Herman *et al*., 1996), using the EZ DNA Methylation Gold kit (Zymo Research, Irvine, CA, USA), according to the manufacturer's instructions. If the amount of DNA was < 500 ng, salmon sperm DNA was added up to a total of 500 ng. All bisulphite‐converted DNA was purified and collected in 40 μL of Tris–EDTA solution.

### 2.5 Droplet digital methylation‐specific PCR

Duplex ddPCR was designed for each gene locus by including two double‐quenched hydrolysis probes: one FAM‐labelled probe to recognize the nonmethylated sequence; and one HEX‐labelled probe to recognize the methylated sequence. Both probes were designed to anneal the same promoter region of each gene. To improve precision, double‐quenched probes (having a 3'IBFQ quencher and an internal ZEN quencher) were produced. Oligonucleotide and probe sequences are listed in Table S1 along with amplification conditions. Twenty microliter was used as the final volume per reaction. For each reaction, 10 µL of 2× Probes ddPCR Supermix (No dUTP; Bio‐Rad Laboratories, Hercules, CA, USA), 0.4 µL of primers (final concentration of 400 nm each), 0.2 µL of probes (final concentration of 200 nm each), and 1 µL of bisulphite‐treated DNA were used.

Each assay was performed in duplicate. The ddPCR assays were performed on a QX‐200 ddPCR System (Bio‐Rad Laboratories) as previously described (Ferracin *et al*., 2016). The amplification conditions were as follows: 95 °C for 10 min, 40 cycles at 94 °C for 30 s, annealing temperature (*T*_a_) for 1 min (see Table S1 for the *T*_a_ of each locus‐specific reaction), followed by 10 min at 98 °C and a final hold at 4 °C. All ramping rates were reduced to 2 °C per second. After the amplification, the ddPCR assay was assessed by the droplet reader (Bio‐Rad Laboratories to analyse each droplet using a two‐colour detection system. The number of positive and negative droplets for each fluorophore (FAM and HEX) in each sample was counted with quantasoft software (Bio‐Rad Laboratories). Poisson distribution of the positive droplets was used to determine the absolute quantification of samples targets (initial copy number of each methylated gene) expressed as copies·µL^−1^. A result was considered positive when both duplicates exhibited the presence of HEX‐positive droplets.

Since DNA isolated from BW samples largely originates from exfoliated normal cells, the quantification of nonmethylated DNA by FAM‐labelled probes represented an internal quality control check and a quantification of the isolated DNA. The detection of at least 200 FAM‐positive droplets was required for the further analysis of the sample. The eventual absence of HEX‐labelled droplets was considered truly negative only in the presence of > 200 FAM‐positive droplets (limit of detection, LOD = 0.5%). In most cases, we obtained 1000–2000 FAM‐positive droplets (LOD = 0.05–0.1%; an example is shown in Fig. S1), which allowed us to readily assess the presence/absence of aberrant methylation at the *RASSF1A*, *PRPH*, *DLC1*, and *CDH1* loci.

### 2.6 Cancer‐gene mutation analyses by NGS

Amplicon libraries were prepared using the Oncomine Lung Cell‐Free Total Nucleic Acid assay (Thermo Fisher Scientific) from 20 to 50 ng of total DNA/RNA isolated from BW samples. To identify the sample, each library was barcoded with a unique oligonucleotide identifier, according to the manufacturer's instructions. Libraries were pooled together in groups of 24/chip (Ion 540) and sequenced on the Ion S5 System (Thermo Fisher Scientific), achieving an average sequencing depth of 7000× (molecular coverage) and average reads number of 3 500 000 for sample. Sequencing raw‐data analysis was performed using torrent suite v. 5.10.1 and ion reporter 5.10.5 (Thermo Fisher Scientific). Briefly, low‐quality reads were removed, adapter sequences trimmed and samples sequence aligned against a reference genome (hg19) using the Torrent Mapping Alignment Program (Thermo Fisher, Carlsbad, CA, USA). Subsequently, the aligned BAM files were uploaded to Ion Reporter and processed using the *ad hoc* Oncomine TagSeq Lung v2 Liquid Biopsy w2.1 (Thermo Fisher)—Single Sample workflow. Each sample was analysed for mutations in the *ALK*, *BRAF*, *EGFR*, *ERBB2*, *KRAS*, *MAP2K1*, *MET*, *NRAS*, *PIK3CA*, *ROS1,* and *TP53* genes and for *ALK*, *ROS1*, and *RET* gene fusions. Next‐generation sequencing (NGS) raw data are available in the European Nucleotide Archive (https://www.ebi.ac.uk/ena) under the accession number PRJEB38273.

### 2.7 Statistical analyses

The presence or absence of methylation (discrete category variables) was analysed with Fisher's exact test. A *P*‐value < 0.05 was considered significant. Diagnostic accuracy of each marker, alone or in combination with others, was also evaluated. The diagnostic value of a marker was calculated as the number of correct answers versus the total number of samples analysed. Specifically, the value was obtained from the ratio between the true positives (lung cancer patients positive for methylation) and true negatives (noncancer patients negative for methylation) out of the total cases analysed.

## 3 Results

### 3.1 DNA methylation biomarkers for lung cancer in bronchial washings

After investigating tens of potential methylation biomarkers in lung cancer samples (data not shown), we selected the CpG islands of the *CDH1*, *RASSF1A*, *PRPH* and *DLC1* gene promoters, because they displayed aberrant methylation in more than 50% of lung cancer samples and no aberrant methylation in normal tissues. We examined the methylation status of these CpG islands in BWs from 129 nonselected consecutive patients undergoing FOB.

Malignant cancer was diagnosed in 98 patients: 91 had a lung carcinoma and seven had lung metastases due to other types of neoplasm (mostly colorectal cancer). Aberrant DNA methylation was detected in six of the seven BW samples from patients with metastatic lesions, and albeit of potential interest, they formed a group that was too small to reach any significant conclusion. In addition, since the study was designed to investigate lung cancer, data from patients with metastases in the lung were excluded from the final statistical analyses to avoid the risk of potential data distortion. The remaining 31 patients had nonmalignant disorders, so were used as negative controls (see Table 1): the definitive diagnoses for these negative controls were obtained by histo‐pathological assessments as well as by proven lesion reduction/resolution or stability for at least 12 months upon clinical/radiological follow‐up.

**Fig. 1:**
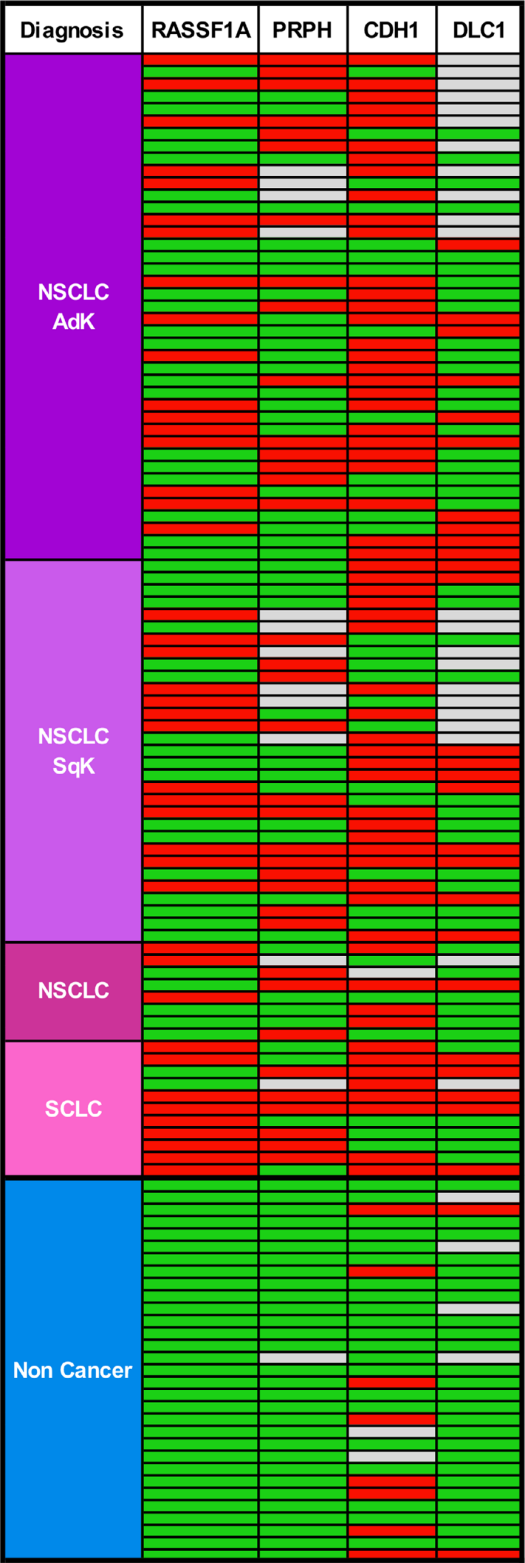
Biomarker methylation profile in BW fluid. Red boxes indicate the presence of methylated DNA, green boxes indicate nonmethylated DNA, and grey boxes indicate either not analysed samples or uninformative results. noncancer, samples from control patients; NSCLC, nonsmall cell lung cancer; SCLC, small cell lung cancer.

Methylation status was analysed by two‐colour ddMSP. Aberrant methylation in at least one marker was present in 88 of 91 (97%) lung cancer patients. In samples from noncancer patients, *RASSF1A*, *PRPH* and *DLC1* exhibited excellent specificity, whereas methylation at *CDH1* was detected in eight of 29 (28%) cases (Fig. 1).

### 3.2 Diagnostic value of our four‐gene DNA methylation panel

**Fig. 2:**
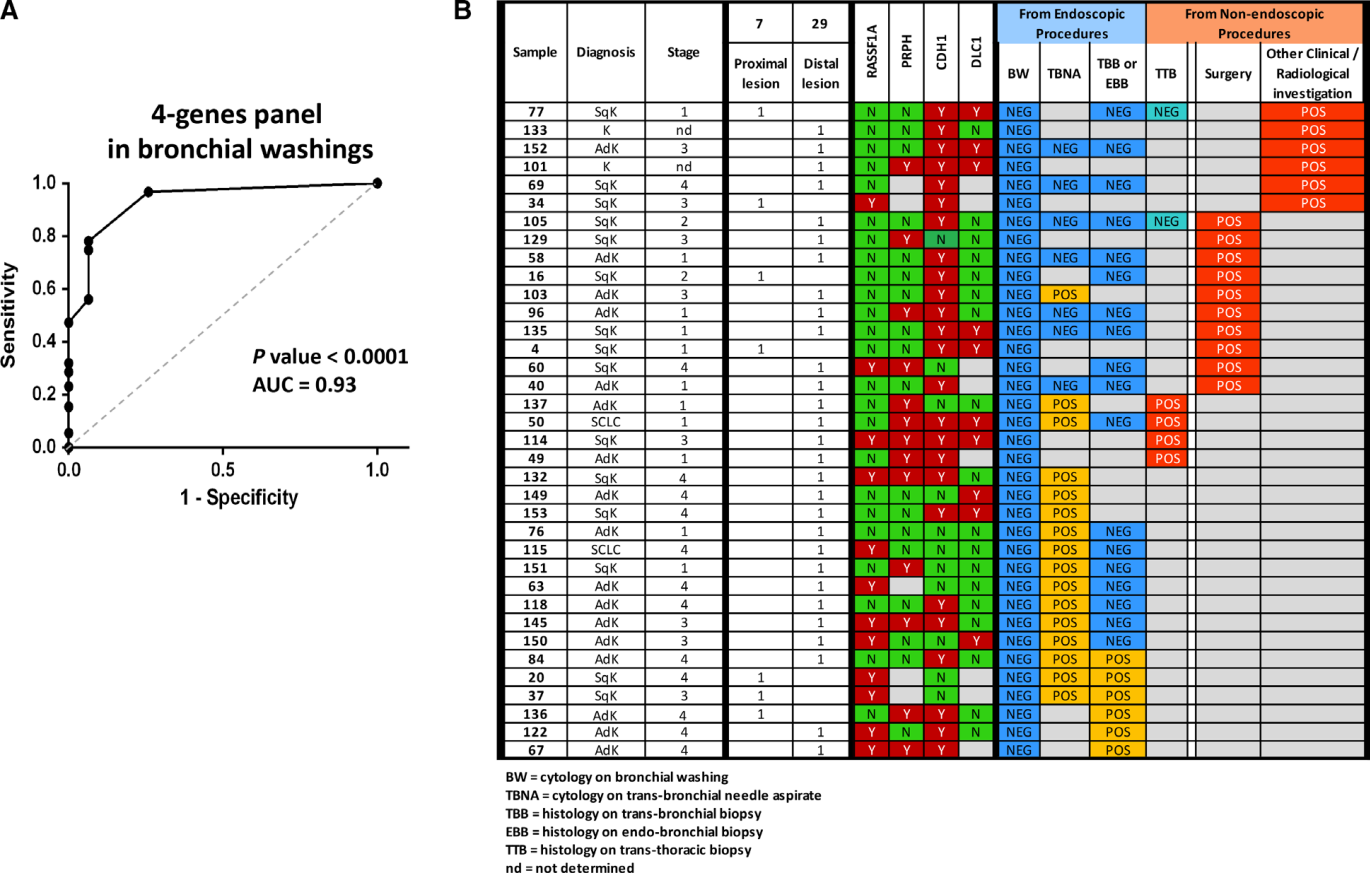
Diagnostic performance of the four‐gene panel in BW samples. (A) Receiver operating characteristic curve analysis, as described in the text. AUC, area under curve. The *P*‐value of the ROC curve was automatically calculated by the prism software (www.graphpad.com) to assess how close to 0.50, the null hypothesis, is the AUC. (B) The 36 lung cancer lesions missed by cytological analysis of BW samples. Twenty‐nine were from peripheral lesions. In these cases, the definitive diagnosis was achieved by transbronchial needle aspiration (TBNA) biopsy, transbronchial or endobronchial biopsy (TBB or EBB), transthoracic biopsy (TTB), or surgical intervention. In six cases, the diagnosis was based on other medical evidence. In 35 (97%) of these BW samples, the 4‐gene panel assay was positive for at least one of the markers (Y = positive; N = negative; empty (grey box) = not tested or uninformative results).

By matching methylation results with final diagnoses, aberrant methylation at each locus was significantly associated with cancer (Table 2). For diagnostic purposes, the best result was achieved by the inclusion of *RASSF1A*, *PRPH*, *DLC1* and *CDH1* in a panel. The panel had a sensitivity of 97% and a specificity of 74% (overall diagnostic accuracy, 0.88). Positivity of the panel conferred a relative risk of 7.3, with a diagnostic odds ratio of 76.1 (95% CI, 18.6–312; Table 2). To perform receiver operating characteristic (ROC) curve analysis, we assigned a specific strength to each methylation marker on the basis of its own specificity (*RASSF1A* = 1.00; *PRPH* = 1.00; *DLC1* = 0.94; *CDH1* = 0.74), and then for each patient, the diagnostic strength was calculated as the sum of the four‐gene panel results. ROC curve analysis on these data gave an area under the curve of 0.93 and confirmed the excellent diagnostic power of the four‐gene panel (Fig. 2A).

**Table 2:** Assessment of diagnostic value of DNA methylation markers. CI, confidence interval.

Test	Sample type	Disease + (*n*)	Controls (*n*)	Fisher's exact test *P*‐value (two‐sided)	Sensitivity	Specificity	Relative risk (95% CI)^a^	Diagnostic odds ratio (95% CI)^a^	Diagnostic accuracy (%)	Positive predictive value (95% CI)	Negative predictive value (95% CI)
Cytology	BW	90	31	< 0.0001	0.59 (0.49–0.69)	1.00 (0.89–1.00)	1.84 (1.48–2.28)	89.9 (5.3–1516)	69	1.00 (0.93–1.00)	0.46 (0.33–0.58)
CDH1	BW	90	31	0.0001	0.64 (0.54–0.74)	0.74 (0.55–0.88)	1.51 (1.19–1.92)	5.2 (2.1–13.0)	68	0.88 (0.77–0.94)	0.42 (0.29–0.56)
DLC1	BW	69	27	0.0027	0.37 (0.26–0.48)	0.94 (0.79–0.99)	1.48 (1.22–1.79)	8.4 (1.9–37.8)	53	0.94 (0.79–0.99)	0.37 (0.26–0.48)
PRPH	BW	79	30	< 0.0001	0.40 (0.30–0.51)	1.00 (0.89–1.00)	1.57 (1.34–1.85)	42.2 (2.5–712)	56	1.00 (0.90–1.00)	0.36 (0.27–0.48)
RASSF1A	BW	91	31	< 0.0001	0.46 (0.35–0.56)	1.00 (0.89–1.00)	1.63 (1.37–1.94)	52.8 (3.1–890)	60	1.00 (0.91–1.00)	0.39 (0.28–0.50)
RASSF1A/ CDH1/ PRPH/ DLC1	BW	91	31	< 0.0001	0.97 (0.91–0.99)	0.74 (0.55–0.88)	7.33 (2.54–21.2)	76.1 (18.6–312)	88	0.92 (0.84–0.96)	0.87 (0.68––0.97)

^a^ Since the values of the contingency table included a zero, the odds ratio was calculated by adding 0.5 to each value of the table.

Bronchial washing samples, especially in the case of distal or peripheral lesions, are often inadequate for efficient diagnosis: indeed, we were not able to reach a definitive diagnosis in 36 patients (about 40% of our lung cancer patients, and 29 of whom had peripheral lesions) through cytological analysis of their BWs (Fig. 2B). To reach definitive diagnosis in these patients, more‐invasive and potentially risky procedures had to be performed (Fig. 2B). In contrast, analysis of methylation markers produced positive results in 35 of the 36 BW samples (Fig. 2B).

### 3.3 Predictive value of gene mutations assessed on BWs

We analysed DNA and RNA from 73 BW samples from cancer patients and 14 samples from noncancer patients. Results are summarized in Table 3 (details in Table S2). Mutations on *EGFR*, *ERBB2* mutations and *ROS1* fusions were found in patients diagnosed with AdKs, whereas *KRAS*, *PIK3CA* and *TP53* mutations were detected in AdK as well as in squamous carcinoma (SqK) patients. Atypical mutations were found on *BRAF* (p.G469V) in one AdK patient and on *ALK* (p.R1275Q) in one AdK and two SqK patients. Analysis of the 14 noncancer patients revealed the presence of mutations on *KRAS*, *PIK3CA* and *TP53* in nine individuals. The frequent mutation detection in noncancer patients revealed the low specificity of this approach for diagnostic purposes.

**Table 3:** Detection of gene mutations in BWs.

			EGFR	ERBB2/HER2	ROS1 fusions	ROS1 mutations	ALK fusions	ALK mutations ^a^	KRAS	PIK3CA	TP53	BRAF ^b^	MET	MAP2K1	NRAS	RET
NSCLC	AdK	tot	52	52	52	52	52	52	52	52	52	52	52	52	52	52
		mut	4	1	2	0	0	1	15	13	22	1	0	0	0	0
		%	7.7	1.9	3.8	0.0	0.0	1.9	28.8	25.0	42.3	1.9	0.0	0.0	0.0	0.0
	SqK	tot	21	21	21	21	21	21	21	21	21	21	21	21	21	21
		mut	0	0	0	0	0	2	3	9	14	0	0	0	0	0
		%	0.0	0.0	0.0	0.0	0.0	9.5	14.3	42.9	66.7	0.0	0.0	0.0	0.0	0.0
Non tumour patients		tot	14	14	14	14	14	14	14	14	14	14	14	14	14	14
		mut	0	0	0	0	0	1	2	4	4	0	0	0	0	0
		%	0.0	0.0	0.0	0.0	0.0	7.1	14.3	28.6	28.6	0.0	0.0	0.0	0.0	0.0

^a^ ALK mutation = p.R1275Q.

^b^ BRAF mutation = p.G469V.

In contrast, predictive value was valuable. To assess the predictive value of mutation analyses, we compared the results obtained from BWs with those from tumour biopsies. The comparison was only possible in the 52 AdK patients, since, as required by clinical guidelines, mutations in tumour samples are only investigated in that histological subtype. Details of tissue versus BWs are reported in Table S3 and summarized in Table 4. Where mutation data from tissue samples were available, the data from BWs revealed to be in good concordance with the matched tumour samples. The few differences included an *EGFR* mutation and an *ALK* fusion detected by IHC on tissue biopsies but missed in BWs, and two *KRAS* mutations detected in BWs but missed in tissue biopsies. A number of mutation analyses could only be performed on BWs, evidencing the presence of two additional *EGFR* mutations, seven *KRAS* mutations, and two different *ROS1* fusions. Several mutations were also found on *PIK3CA* and *TP53*. These findings indicate that detection of actionable mutations is feasible for BWs. In addition to the above‐described concordance between BW analysis and tumour tissue mutational results, NGS on BWs has the advantage that it can be performed on all patients, including those for whom tumour tissue was missed, allowing the discovery of additional mutations eventually on actionable genes.

**Table 4:** Concordance of mutations between tissue and BW in adenocarcinoma patients.

		Tissue	BW	Concordance (%)	Additional BWs
ALK fusions	Mut	1	0	96	0
	WT	27	28		24
BRAF V600E	Mut	0	0	100	1
	WT	7	7		44
EGFR	Mut	3	2	97	2
	WT	29	30		18
ERBB2/HER2	Mut	1	1	100	0
	WT	5	5		46
KRAS	Mut	5	7	90	7
	WT	15	13		25
MET	Mut	0	0	100	0
	WT	6	6		46
ROS1 fusions	Mut	0	0	100	2
	WT	15	15		35

## 4 Discussion

At initial diagnosis, endoscopic FOB is the routinely used approach to obtain the biological material needed to perform cyto‐ or histo‐pathological analyses. Although BW is a low‐risk method for the collection of such samples, depending on the location of the primary tumour this technique is not always adequate for cytological diagnostic purposes. Thus, in about 40% of cases, more‐invasive approaches are needed to obtain biopsy material. To address this issue, molecular investigations on BWs could represent useful alternatives or additions for improving diagnostic performance and/or reducing the need of potentially risky procedures. Li and collaborators analysed mutations in BALs, finding that the combined detection of mutations on *KRAS* and *TP53* yielded a sensitivity of 66% for the diagnosis of peripheral nonsmall cell lung cancer (Li *et al*., 2014). However, current guidelines do not recommend BAL as a routine approach for the diagnosis of peripheral lung lesions. Conversely, BW is a routinely employed, less‐invasive procedure. Thus, in the present work, we focused on BW, which, despite theoretically having a lower power for diagnosing peripheral lesions, is a procedure better tolerated by all patients. Founded on the diagnostic yield of methylation assays and the high concordance rate of mutational analysis as compared with pathology results, our findings demonstrate that BW specimens are suitable for diagnostic purposes, even for peripheral lesions.

In the present study, we investigated the use of DNA and RNA isolated from BW samples, evaluating diagnostic potential and predictive value. Our results indicate that BWs collected during FOB are excellent sample materials on which to generate useful information for diagnostic workup and predictive therapy indications based on molecular examinations. The employed methodological approaches, based on either ddMSP or NGS, exhibited an analytical sensitivity of 0.1% or lower, which is adequate to recognize the traces of nucleic acids originating from cancer cells.

For diagnostic purposes, we analysed tumour‐specific aberrant DNA methylation with a 4‐gene panel made up of *CDH1*, *PRPH*, *RASSF1A* and *DLC1*. Methylation at *RASSF1A* and *CDH1* loci has been previously investigated in human lung cancer, either for their clinical pathological significance (Brock *et al*., 2008; Yu *et al*., 2015) or for their potential use as tumour biomarkers (Baryshnikova *et al*., 2008; Han *et al*., 2009; Hubers *et al*., 2014a; Hubers *et al*., 2014b; Hubers *et al*., 2015). In particular, *RASSF1A* methylation has been utilized because of its high specificity and early appearance in tumour development. By correlating the methylation results with final diagnoses, each locus exhibited very good specificity. Aberrant methylation on *RASSF1A* or *PRPH* exhibited 100% specificity, and on *DLC1* an excellent 94% specificity. Only *CDH1* exhibited less‐than‐optimal specificity with a value around 74%. Thus, our findings show that the assessment of aberrant DNA methylation on BW fluids could be a useful aid for initial lung cancer diagnosis. Nevertheless, although the *RASSF1A*, *PRPH*, *CDH1* and *DLC1* DNA methylation panel is highly sensitive and specific, it requires further improvement with the addition of other highly specific biomarkers.

More importantly, the four‐gene panel achieved excellent diagnostic accuracy: overall, the panel exhibited 88% diagnostic accuracy, 97% sensitivity and, largely because of *CDH1*, 74% specificity (RR, 7.3; diagnostic OR, 76.1). This diagnostic accuracy is greater than that obtained through traditional cytological analyses. Significantly, for the 36 BW samples from cancer patients that were inadequate for reaching a cytologically based diagnosis, results from methylation analysis provided a diagnostic indication of cancer in 35 (97%) of them, a result that could have potentially avoided more‐invasive interventions for these patients.

Most of the panel genes are not only tumour biomarkers, but are also pathogenically important. Loss of expression of *CDH1*‐, *RASSF1A*‐ and *DLC1*‐encoded proteins is relevant for pathogenic mechanisms that promote cell motility and metastatic potential. *CDH1* encodes a calcium‐dependent cell adhesion protein [cadherin 1, type 1, E‐cadherin (epithelial)] whose loss can contribute to the metastatic potential of cancer cells (Kim *et al*., 2016). The protein encoded by *RASSF1A* binds to activated RAS to mediate apoptosis (Donninger *et al*., 2007; Gordon and Baksh, 2011; Grawenda and O'Neill, 2015). The protein encoded by *DLC1* interacts with the DNA repair proteins XPA and BRCA2; it also inhibits the accumulation of cyclin D1 and induces cell cycle arrest. *DLC1* (deleted in liver cancer 1) encodes for a GTPase‐activating protein (GAP) member of the rho‐GAP family of proteins: it can stop the signalling of RHOA, RHOB, RHOC and CDC42. Loss of *DLC1* expression results in the constitutive activation of the rho‐GTPases, which promote increased cell motility and changes in morphology (Barras and Widmann, 2014; Popescu and Goodison, 2014). The role of *PRPH* in cancer is less clear: it encodes the cytoskeletal protein peripherin, a type III intermediate filament with homology to other cytoskeletal proteins such as desmin, and that is found in neurons of the peripheral nervous system. To date, aberrant *PRPH* methylation has been reported in two studies on liver cancer and neuroblastoma (Decock *et al*., 2012; Revill *et al*., 2013).

We also give evidence on the value of DNA/RNA analysis of BW specimens to direct therapeutic decisions. The use of highly sensitive approaches, such as ddPCR or NGS, is already being employed to detect traces of nucleic acids originating from tumour cells in liquid biopsies: they are performed mainly on plasma samples to monitor the patient's response to therapy. The present study highlights the possible use of BWs to provide predictive indications during a very early diagnostic phase. However, whether anticipating the start of treatment translates into clinically measurable benefits cannot be assessed here, as this would require an *ad hoc* trial.

When BW results were matched to molecular data obtained from AdK samples, the concordance was almost complete, with only one *EGFR* mutation and an *ALK* fusion missed. Most notably, in cases in which tumour tissues could not be analysed, additional mutations affecting the actionable genes *EGFR* and *ROS1* were detected in BW samples. We also detected the atypical lung cancer mutations *BRAF* (p.G469V) and *ALK* (p.R1275Q), whose clinical significance should be further evaluated. Although at present the significance of these mutations in terms of response to target therapies is not known, the *ALK* mutation c.3824G>A (p.R1275Q), albeit infrequent in lung cancer, is commonly found in neuroblastoma, where this mutation covers about one third of all *ALK* mutations, which affect about 8–9% of the cases (Tate *et al*., 2019). The response to crizotinib or other ALK inhibitors is presently unknown, but preclinical studies have shown that R1275Q cell lines are sensitive to crizotinib (Bresler *et al*., 2014; Schonherr *et al*., 2011), and clinical trials investigating crizotinib in neuroblastoma are underway. Taken together, the findings of this study on aberrant methylation and cancer‐gene mutations strongly suggest that molecular analysis of BWs can indeed be part of the complete diagnostic and predictive workup in the very early phases of patient management.

In addition, although not specifically investigated in this study, the described approaches are potentially relevant for the early diagnosis of lung cancer. Early diagnosis, along with measures of primary prevention, could be effective in improving prognosis and reducing mortality due to lung cancer (Fleischhacker *et al*., 2013; Hubers *et al*., 2013; Langevin *et al*., 2015). A number of guidelines and recommendations for lung cancer screening exist (Bach *et al*., 2012; Jaklitsch *et al*., 2012; Smith *et al*., 2015). Individuals at risk of lung cancer have been recognized: these are aged 55–74 years old with a ≥30 packs/year smoking history, either currently smoking or who have quit within the past 15 years. However, no safe screening programme for the early detection of lung cancer is widely available yet (Kubik and Polak, 1986; Melamed *et al*., 1984). Low‐dose spiral computerized tomography has been suggested as a modality for lung cancer screening (Aberle *et al*., 2011), but limitations have been reported (Aberle *et al*., 2013). Thus, the need for more‐sensitive and specific approaches still exists. Can molecular investigations find application in screening programmes for the early detection of lung cancer? A number of studies have suggested the use of circulating microRNAs (Montani *et al*., 2015; Sozzi *et al*., 2014; Wang *et al*., 2015; Wozniak *et al*., 2015). Here, we show evidence that the analysis of the methylation status of a small gene panel has a very high diagnostic potential and could be used on BWs obtained periodically from individuals at risk.

Surprisingly, the present study identified mutations in cancer genes in several cancer‐free individuals. This finding is in line with recent studies reporting the presence of cancer‐gene mutations in different noncancer tissues (Lee‐Six *et al*., 2019; Martincorena and Campbell, 2015; Martincorena *et al*., 2018). Here, we identified mutations on *PIK3CA*, *TP53*, *KRAS* and *ALK* in individuals without cancer. *ALK* R1275Q, as discussed above, has an uncertain pathogenic significance in lung cancer. Similarly, the pathogenic significance of *PIK3CA* is also uncertain, judging from the unusually infrequent detection of mutations in lung cancer (2% in adenocarcinoma and 5% in SqKs). Conversely, *TP53* is very frequently affected by mutations (40% in adenocarcinoma, 60% in SqK and 63% in small cell lung cancer), but it generally requires a double hit to become oncogenic. In our case series, most patients are or were heavy smokers, and mutations could have randomly accumulated on several genes in several cells. It should also be noted that these mutations are generally detected at a very low level, suggesting that they occur in a few disparate cells of the lung. Especially for *TP53* mutations, the requirement of two inactivating mutations in the same cell suggests that the detected mutations might represent single hits. We speculate that these single‐hit mutations might confer an increased risk of developing cancer, but are insufficient to promote cancer initiation. Mutations in cancer genes, such as *TP53*, possibly represent molecular evidence of the increased risk of cancer that exists in all smokers. From a practical point of view, our findings indicate that the detection of point mutations on *PIK3CA*, *TP53*, and *ALK* in BWs have limited diagnostic power for lung cancer.

There were also two cases with *KRAS* mutations: a p.G12V mutation detected at 0.16% in patient C_059 and a p.G12D mutation at 3.3% in patient R_023. The first patient never developed a malignant lung lesion, suggesting that no additional alteration able to cooperate with the activated RAS was acquired. It is well established that multiple genetic/epigenetic changes are needed to promote a malignant phenotype. In the second patient, who incidentally exhibited a higher level of the mutant *KRAS* allele, a lung metastasis from a colon adenocarcinoma was diagnosed 3 years later. These findings suggest that the level of variants detected in cancer genes, especially if higher than 1%, should not be disregarded and a surveillance programme should be considered for these patients.

Alterations in certain other genes, namely *EGFR*, *HER2* and *ROS1*, were instead only found in patients with cancer. Albeit relatively infrequent, these mutations might have diagnostic value. In support of this hypothesis, we observed that patient B_203 developed an lung adenocarcinoma about 1 year after an initially negative diagnosis, but a mutation on *EGFR* was already detected in the initial BW analysis. Although just a single case, this example suggests that the presence of mutations in cancer genes might identify individuals carrying a different risk of developing lung cancer, which puts forward the case, as indicated above, for the development of specific surveillance programmes for these individuals. To investigate this hypothesis, the present study suggests that molecular analyses conducted on BWs taken from at‐risk individuals should be thoroughly investigated through trials based on extensive case studies and accurate follow‐up.

## 5 Conclusions

Our study demonstrates that the use of BWs for molecular analyses is feasible. Methylation and gene mutation analyses could be performed to support and complete the current clinical diagnostic/predictive strategies.

## Conflict of interest

The authors declare no conflict of interest.

## Author contributions

RR and LL performed targeted sequencing experiments and edited the manuscript; EM performed methylation experiments and statistical analyses; ES, SM, LM, DR provided clinical samples and data; CB performed bioinformatics analyses; DR, EC, VC, RR, GL, RG, AF provided clinical data and pathology review of the samples; AP, SS, FR, GLC and MN performed the study design, wrote and proofed the manuscript.

## Supporting information


**Fig. S1.** Typical 1D and 2D plots for analysis of droplet fluorescence in RASSF1A methylation assay.

**Table S1.** ddPCR oligonucleotide and probe sequences, annealing temperatures and size products.

**Table S2.** Mutation analyses in bronchial washings from cohort study patients.

**Table S3.** Mutation analyses in bronchial washings and tissue biopsies from NSCLC adenocarcinoma patients.

## Data Availability

All data are available in supplementary files. NGS row data will be uploaded in European Nucleotide Archive (ENA).

## References

[mol212713-bib-0001] Aberle DR , Abtin F and Brown K (2013) Computed tomography screening for lung cancer: has it finally arrived? Implications of the national lung screening trial. J Clin Oncol 31, 1002–1008. 23401434 10.1200/JCO.2012.43.3110 PMC3589698

[mol212713-bib-0002] Aberle DR , Adams AM , Berg CD , Black WC , Clapp JD , Fagerstrom RM , Gareen IF , Gatsonis C , Marcus PM and Sicks JD (2011) Reduced lung‐cancer mortality with low‐dose computed tomographic screening. N Engl J Med 365, 395–409. 21714641 10.1056/NEJMoa1102873 PMC4356534

[mol212713-bib-0003] Bach PB , Mirkin JN , Oliver TK , Azzoli CG , Berry DA , Brawley OW , Byers T , Colditz GA , Gould MK , Jett JR *et al* (2012) Benefits and harms of CT screening for lung cancer: a systematic review. JAMA 307, 2418–2429. 22610500 10.1001/jama.2012.5521 PMC3709596

[mol212713-bib-0004] Barras D and Widmann C (2014) GAP‐independent functions of DLC1 in metastasis. Cancer Metastasis Rev 33, 87–100. 24338004 10.1007/s10555-013-9458-0

[mol212713-bib-0005] Baryshnikova E , Destro A , Infante MV , Cavuto S , Cariboni U , Alloisio M , Ceresoli GL , Lutman R , Brambilla G , Chiesa G *et al* (2008) Molecular alterations in spontaneous sputum of cancer‐free heavy smokers: results from a large screening program. Clin Cancer Res 14, 1913–1919. 18347195 10.1158/1078-0432.CCR-07-1741

[mol212713-bib-0006] Belinsky SA , Klinge DM , Dekker JD , Smith MW , Bocklage TJ , Gilliland FD , Crowell RE , Karp DD , Stidley CA and Picchi MA (2005) Gene promoter methylation in plasma and sputum increases with lung cancer risk. Clin Cancer Res 11, 6505–6511. 16166426 10.1158/1078-0432.CCR-05-0625

[mol212713-bib-0007] Belinsky SA , Liechty KC , Gentry FD , Wolf HJ , Rogers J , Vu K , Haney J , Kennedy TC , Hirsch FR , Miller Y *et al* (2006) Promoter hypermethylation of multiple genes in sputum precedes lung cancer incidence in a high‐risk cohort. Cancer Res 66, 3338–3344. 16540689 10.1158/0008-5472.CAN-05-3408

[mol212713-bib-0008] Bresler SC , Weiser DA , Huwe PJ , Park JH , Krytska K , Ryles H , Laudenslager M , Rappaport EF , Wood AC , McGrady PW *et al* (2014) ALK mutations confer differential oncogenic activation and sensitivity to ALK inhibition therapy in neuroblastoma. Cancer Cell 26, 682–694. 25517749 10.1016/j.ccell.2014.09.019 PMC4269829

[mol212713-bib-0009] Brock MV , Hooker CM , Ota‐Machida E , Han Y , Guo M , Ames S , Glockner S , Piantadosi S , Gabrielson E , Pridham G *et al* (2008) DNA methylation markers and early recurrence in stage I lung cancer. N Engl J Med 358, 1118–1128. 18337602 10.1056/NEJMoa0706550

[mol212713-bib-0010] Contoli M , Gnesini G , Artioli D , Ravenna C , Sferra S , Romanazzi C , Marangoni E , Guzzinati I , Pasquini C , Papi A *et al* (2013) Midazolam in flexible bronchoscopy premedication: effects on patient‐related and procedure‐related outcomes. J Bronchology Interv Pulmonol 20, 232–240. 23857197 10.1097/LBR.0b013e3182a10b7a

[mol212713-bib-0011] Decock A , Ongenaert M , Hoebeeck J , De Preter K , Van Peer G , Van Criekinge W , Ladenstein R , Schulte JH , Noguera R , Stallings RL *et al* (2012) Genome‐wide promoter methylation analysis in neuroblastoma identifies prognostic methylation biomarkers. Genome Biol 13, R95. 23034519 10.1186/gb-2012-13-10-r95 PMC3491423

[mol212713-bib-0012] Donninger H , Vos MD and Clark GJ (2007) The RASSF1A tumor suppressor. J Cell Sci 120, 3163–3172. 17878233 10.1242/jcs.010389

[mol212713-bib-0013] Du Rand IA , Blaikley J , Booton R , Chaudhuri N , Gupta V , Khalid S , Mandal S , Martin J , Mills J , Navani N *et al* (2013) British Thoracic Society guideline for diagnostic flexible bronchoscopy in adults: accredited by NICE. Thorax 68(Suppl 1), i1–i44. 23860341 10.1136/thoraxjnl-2013-203618

[mol212713-bib-0014] Ferracin M , Salamon I , Lupini L , Miotto E , Sabbioni S and Negrini M (2016) Circulating MicroRNA quantification using DNA‐binding dye chemistry and droplet digital PCR. J Vis Exp e54102. 10.3791/54102 PMC4993260 27403944

[mol212713-bib-0015] Fleischhacker M , Dietrich D , Liebenberg V , Field JK and Schmidt B (2013) The role of DNA methylation as biomarkers in the clinical management of lung cancer. Expert Rev Respir Med 7, 363–383. 23964627 10.1586/17476348.2013.814397

[mol212713-bib-0016] Gordon M and Baksh S (2011) RASSF1A: not a prototypical Ras effector. Small GTPases 2, 148–157. 21776416 10.4161/sgtp.2.3.16286 PMC3136945

[mol212713-bib-0017] Grawenda AM and O'Neill E (2015) Clinical utility of RASSF1A methylation in human malignancies. Br J Cancer 113, 372–381. 26158424 10.1038/bjc.2015.221 PMC4522630

[mol212713-bib-0018] Han W , Wang T , Reilly AA , Keller SM and Spivack SD (2009) Gene promoter methylation assayed in exhaled breath, with differences in smokers and lung cancer patients. Respir Res 10, 86. 19781081 10.1186/1465-9921-10-86 PMC2759916

[mol212713-bib-0019] Herman JG , Graff JR , Myohanen S , Nelkin BD and Baylin SB (1996) Methylation‐specific PCR: a novel PCR assay for methylation status of CpG islands. Proc Natl Acad Sci USA 93, 9821–9826. 8790415 10.1073/pnas.93.18.9821 PMC38513

[mol212713-bib-0020] Hubers AJ , Brinkman P , Boksem RJ , Rhodius RJ , Witte BI , Zwinderman AH , Heideman DA , Duin S , Koning R , Steenbergen RD *et al* (2014a) Combined sputum hypermethylation and eNose analysis for lung cancer diagnosis. J Clin Pathol 67, 707–711. 24915850 10.1136/jclinpath-2014-202414

[mol212713-bib-0021] Hubers AJ , Heideman DA , Burgers SA , Herder GJ , Sterk PJ , Rhodius RJ , Smit HJ , Krouwels F , Welling A , Witte BI *et al* (2015) DNA hypermethylation analysis in sputum for the diagnosis of lung cancer: training validation set approach. Br J Cancer 112, 1105–1113. 25719833 10.1038/bjc.2014.636 PMC4366885

[mol212713-bib-0022] Hubers AJ , Prinsen CF , Sozzi G , Witte BI and Thunnissen E (2013) Molecular sputum analysis for the diagnosis of lung cancer. Br J Cancer 109, 530–537. 23868001 10.1038/bjc.2013.393 PMC3738145

[mol212713-bib-0023] Hubers AJ , van der Drift MA , Prinsen CF , Witte BI , Wang Y , Shivapurkar N , Stastny V , Bolijn AS , Hol BE , Feng Z *et al* (2014b) Methylation analysis in spontaneous sputum for lung cancer diagnosis. Lung Cancer 84, 127–133. 24598366 10.1016/j.lungcan.2014.01.019

[mol212713-bib-0024] Jaklitsch MT , Jacobson FL , Austin JH , Field JK , Jett JR , Keshavjee S , MacMahon H , Mulshine JL , Munden RF , Salgia R *et al* (2012) The American Association for Thoracic Surgery guidelines for lung cancer screening using low‐dose computed tomography scans for lung cancer survivors and other high‐risk groups. J Thorac Cardiovasc Surg 144, 33–38. 22710039 10.1016/j.jtcvs.2012.05.060

[mol212713-bib-0025] Kim SA , Inamura K , Yamauchi M , Nishihara R , Mima K , Sukawa Y , Li T , Yasunari M , Morikawa T , Fitzgerald KC *et al* (2016) Loss of CDH1 (E‐cadherin) expression is associated with infiltrative tumour growth and lymph node metastasis. Br J Cancer 114, 199–206. 26742007 10.1038/bjc.2015.347 PMC4815802

[mol212713-bib-0026] Kubik A and Polak J (1986) Lung cancer detection. Results of a randomized prospective study in Czechoslovakia. Cancer 57, 2427–2437. 3697941 10.1002/1097-0142(19860615)57:12<2427::aid-cncr2820571230>3.0.co;2-m

[mol212713-bib-0027] Langevin SM , Kratzke RA and Kelsey KT (2015) Epigenetics of lung cancer. Transl Res 165, 74–90. 24686037 10.1016/j.trsl.2014.03.001 PMC4162853

[mol212713-bib-0028] Lee‐Six H , Olafsson S , Ellis P , Osborne RJ , Sanders MA , Moore L , Georgakopoulos N , Torrente F , Noorani A , Goddard M *et al* (2019) The landscape of somatic mutation in normal colorectal epithelial cells. Nature 574, 532–537. 31645730 10.1038/s41586-019-1672-7

[mol212713-bib-0029] Li J , Hu YM , Wang Y , Tang XP , Shi WL and Du YJ (2014) Gene mutation analysis in non‐small cell lung cancer patients using bronchoalveolar lavage fluid and tumor tissue as diagnostic markers. Int J Biol Markers 29, e328–e336. 24519547 10.5301/jbm.5000075

[mol212713-bib-0030] Martincorena I and Campbell PJ (2015) Somatic mutation in cancer and normal cells. Science 349, 1483–1489. 26404825 10.1126/science.aab4082

[mol212713-bib-0031] Martincorena I , Fowler JC , Wabik A , Lawson ARJ , Abascal F , Hall MWJ , Cagan A , Murai K , Mahbubani K , Stratton MR *et al* (2018) Somatic mutant clones colonize the human esophagus with age. Science 362, 911–917. 30337457 10.1126/science.aau3879 PMC6298579

[mol212713-bib-0032] Melamed MR , Flehinger BJ , Zaman MB , Heelan RT , Perchick WA and Martini N (1984) Screening for early lung cancer. Results of the Memorial Sloan‐Kettering study in New York. Chest 86, 44–53. 6734291 10.1378/chest.86.1.44

[mol212713-bib-0033] Molina‐Vila MA , Mayo‐de‐Las‐Casas C , Gimenez‐Capitan A , Jordana‐Ariza N , Garzon M , Balada A , Villatoro S , Teixido C , Garcia‐Pelaez B , Aguado C *et al* (2016) Liquid biopsy in non‐small cell lung cancer. Frontiers in medicine 3, 69. 28066769 10.3389/fmed.2016.00069 PMC5179978

[mol212713-bib-0034] Montani F , Marzi MJ , Dezi F , Dama E , Carletti RM , Bonizzi G , Bertolotti R , Bellomi M , Rampinelli C , Maisonneuve P *et al* (2015) miR‐Test: a blood test for lung cancer early detection. J Natl Cancer Inst 107, djv063. 25794889 10.1093/jnci/djv063

[mol212713-bib-0035] Ofiara LM , Navasakulpong A , Ezer N and Gonzalez AV (2012) The importance of a satisfactory biopsy for the diagnosis of lung cancer in the era of personalized treatment. Curr Oncol 19, S16–S23. 22787407 10.3747/co.19.1062 PMC3377750

[mol212713-bib-0036] Park S , Hur JY , Lee KY , Lee JC , Rho JK , Shin SH and Choi CM (2017) Assessment of EGFR mutation status using cell‐free DNA from bronchoalveolar lavage fluid. Clin Chem Lab Med 55, 1489–1495. 28195541 10.1515/cclm-2016-0302

[mol212713-bib-0037] Popescu NC and Goodison S (2014) Deleted in liver cancer‐1 (DLC1): an emerging metastasis suppressor gene. Mol Diagn Ther 18, 293–302. 24519699 10.1007/s40291-014-0086-3 PMC4032595

[mol212713-bib-0038] Revill K , Wang T , Lachenmayer A , Kojima K , Harrington A , Li J , Hoshida Y , Llovet JM and Powers S (2013) Genome‐wide methylation analysis and epigenetic unmasking identify tumor suppressor genes in hepatocellular carcinoma. Gastroenterology 145, 1424–1435, e1421–1425. 24012984 10.1053/j.gastro.2013.08.055 PMC3892430

[mol212713-bib-0039] Rivera MP and Mehta AC (2007) Initial diagnosis of lung cancer: ACCP evidence‐based clinical practice guidelines. Chest 132, 131S–148S. 17873165 10.1378/chest.07-1357

[mol212713-bib-0040] Schonherr C , Ruuth K , Yamazaki Y , Eriksson T , Christensen J , Palmer RH and Hallberg B (2011) Activating ALK mutations found in neuroblastoma are inhibited by Crizotinib and NVP‐TAE684. Biochem J 440, 405–413. 21838707 10.1042/BJ20101796

[mol212713-bib-0041] Siegel RL , Miller KD and Jemal A (2016) Cancer statistics, 2016. CA Cancer J Clin 66, 7–30. 26742998 10.3322/caac.21332

[mol212713-bib-0042] Smith RA , Manassaram‐Baptiste D , Brooks D , Doroshenk M , Fedewa S , Saslow D , Brawley OW and Wender R (2015) Cancer screening in the United States, 2015: a review of current American cancer society guidelines and current issues in cancer screening. CA Cancer J Clin 65, 30–54. 25581023 10.3322/caac.21261

[mol212713-bib-0043] Sozzi G , Boeri M , Rossi M , Verri C , Suatoni P , Bravi F , Roz L , Conte D , Grassi M , Sverzellati N *et al* (2014) Clinical utility of a plasma‐based miRNA signature classifier within computed tomography lung cancer screening: a correlative MILD trial study. J Clin Oncol 32, 768–773. 24419137 10.1200/JCO.2013.50.4357 PMC4876348

[mol212713-bib-0044] Tate JG , Bamford S , Jubb HC , Sondka Z , Beare DM , Bindal N , Boutselakis H , Cole CG , Creatore C , Dawson E *et al* (2019) COSMIC: the catalogue of somatic mutations in cancer. Nucleic Acids Res 47, D941–D947. 30371878 10.1093/nar/gky1015 PMC6323903

[mol212713-bib-0045] Thiberville L and Salaun M (2010) Bronchoscopic advances: on the way to the cells. Respiration 79, 441–449. 20431326 10.1159/000313495

[mol212713-bib-0046] Topaloglu O , Hoque MO , Tokumaru Y , Lee J , Ratovitski E , Sidransky D and Moon CS (2004) Detection of promoter hypermethylation of multiple genes in the tumor and bronchoalveolar lavage of patients with lung cancer. Clin Cancer Res 10, 2284–2288. 15073103 10.1158/1078-0432.ccr-1111-3

[mol212713-bib-0047] van der Drift MA , van der Wilt GJ , Thunnissen FB and Janssen JP (2005) A prospective study of the timing and cost‐effectiveness of bronchial washing during bronchoscopy for pulmonary malignant tumors. Chest 128, 394–400. 16002962 10.1378/chest.128.1.394

[mol212713-bib-0048] Wang C , Ding M , Xia M , Chen S , Van Le A , Soto‐Gil R , Shen Y , Wang N , Wang J , Gu W *et al* (2015) A Five‐miRNA panel identified from a multicentric case‐control study serves as a novel diagnostic tool for ethnically diverse non‐small‐cell lung cancer patients. EBioMedicine 2, 1377–1385. 26629532 10.1016/j.ebiom.2015.07.034 PMC4634198

[mol212713-bib-0049] Wozniak MB , Scelo G , Muller DC , Mukeria A , Zaridze D and Brennan P (2015) Circulating microRNAs as non‐invasive biomarkers for early detection of non‐small‐cell lung cancer. PLoS One 10, e0125026. 25965386 10.1371/journal.pone.0125026 PMC4428831

[mol212713-bib-0050] Yoneda K , Imanishi N , Ichiki Y and Tanaka F (2019) A liquid biopsy in primary lung cancer. Surg Today 49, 1–14. 29644440 10.1007/s00595-018-1659-2

[mol212713-bib-0051] Yu Q , Guo Q , Chen L and Liu S (2015) Clinicopathological significance and potential drug targeting of CDH1 in lung cancer: a meta‐analysis and literature review. Drug design, development and therapy 9, 2171–2178. 10.2147/DDDT.S78537 PMC4404966 25931811

