# Supplementary material for: Molecular testing on bronchial washings for the diagnosis and predictive assessment of lung cancer
Source: Mol Oncol. 2020 Jun 24;14(9):2163–75. doi: 10.1002/1878-0261.12713 (PMC7463327; doi:10.1002/1878-0261.12713)
Supplement: Supplementary file 1 — Fig. S1. Typical 1D and 2D plots for analysis of droplet fluorescence in RASSF1A methylation assay. [file MOL2-14-2163-s001.pdf]

Molecular testing on bronchial washings for lung cancer diagnostic and predictive assessment

Roberta Roncarati, Laura Lupini, Elena Miotto, Elena Saccenti, Susanna Mascetti, Luca Morandi, Cristian Bassi, Debora Rasio, Elisa Callegari, Valentina Conti, Rosa Rinaldi, Giovanni Lanza, Roberta Gafà, Alberto Papi, Antonio Frassoldati, Silvia Sabbioni, Franco Ravenna, Gian Luca Casoni, and Massimo Negrini.

SUPPORTIVE INFORMATION

Fig. S1. Typical 1D and 2D plots for analysis of droplet fluorescence in RASSF1A methylation assay.

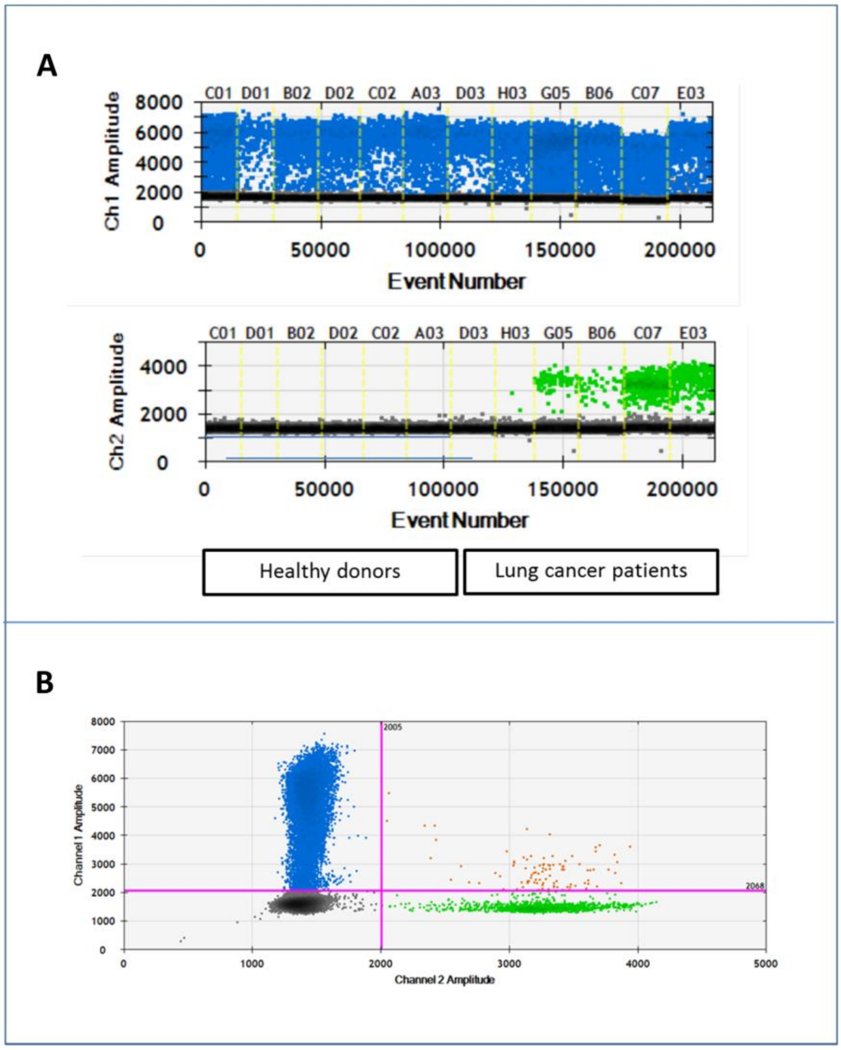

Fig. S1. Typical 1D and 2D plots for analysis of droplet fluorescence in RASSF1A methylation assay. (**A**) 1-D plot: Each droplet from a sample is plotted to show fluorescence intensity vs. droplet number**.** The blue droplets are positive for the FAM probe that detects the non-methylated sequence and each droplet is assigned a value of 1. The green droplets are positive for the HEX probe that detects the methylated sequence and each is assigned a value of 1. The black droplets are negative for both probes (value of 0). Ch1 = channel 1; Ch2 = channel 2. (**B**) 2-D plot: Fluorescence in channel 1 (FAM) is plotted against channel 2 (HEX) for each droplet. The orange droplets are positive for both probes (value of 1 for each probe) and the black droplets are negative for both probes (value of 0 for each probe).
